# Structured, expert-informed selection and harmonization of patient-reported outcome measures (PROMs) in a German multicenter network: the post-COVID syndrome use case

**DOI:** 10.1186/s12955-026-02631-8

**Published:** 2026-09-25

**Authors:** Evelyne Hanc, Katharina Koller, Regina Herold, Luisa Balz, Alexa Kupferschmitt, Deborah Erhart, Jonathan Mang, Bernd Löwe, Volker Köllner, Stephan Frisch, Yesim Erim, Eva Morawa

**Affiliations:** 1 Department of Psychosomatic Medicine and Psychotherapy, University Hospital of Erlangen, Friedrich-Alexander University Erlangen-Nürnberg (FAU), Erlangen, Germany; 2 Department of Neurology, University Hospital Ulm, Ulm, Germany; 3 Department of Psychosomatic Medicine, Research Group Psychosomatic Rehabilitation, Charité – University Medicine Berlin, Berlin, Germany; 4 Rehabilitation Center Seehof, Department of Behavioral Therapy and Psychosomatics, German Federal Pension Insurance, Teltow, Germany; 5 Medical Center for Information and Communication Technology, University Hospital of Erlangen, Friedrich-Alexander University Erlangen-Nürnberg (FAU), Erlangen, Germany; 6 Department of Psychosomatic Medicine and Psychotherapy, University Medical Center Hamburg-Eppendorf, Hamburg, Germany; 7 Department of Psychosomatic Medicine and Psychotherapy, University Hospital Ulm, Ulm University, Ulm, Germany; 8 Post-COVID Center, University Hospital of Erlangen, Erlangen, Germany

**Keywords:** Post-COVID syndrome, Patient-reported outcome measures (PROMs), Symptom recording, Standardization, Clinical care

## Abstract

**Background:**

Patient-reported outcome measures (PROMs) provide subjective information on symptoms, functional ability or quality of life. In post-COVID syndrome (PCS), lacking clear diagnostic criteria or biomarkers, PROMs enable a differentiated assessment of disease course and treatment effects. Within a multicenter research project (EMOPROM LCN), a structured selection process for PROMs was conducted to reach consensus on a validated PROMs battery for PCS, suitable for studies and clinical care, and transferable to other complex conditions.

**Methods:**

An interdisciplinary committee identified key symptoms/syndromes and constructs (e.g., fatigue, depression, post-exertional malaise) and selected PROMs through a structured, expert-based consensus process considering psychometric properties, clinical relevance, feasibility, time efficiency and applicability within the German healthcare context. The questionnaires were organized into thematic domains and compiled into a modular test battery.

**Results:**

The following 13 questionnaires were included in the final PROMs battery: Post-COVID Syndrome Score (PCS-Score), Post-COVID Functional Status Scale (PCFS), Fatigue Scale for Motor and Cognitive Functions (FSMC), Patient Health Questionnaire-9 (PHQ-9), Generalized Anxiety Disorder Scale 7 (GAD-7), DePaul Symptom Questionnaire - Post-exertional malaise (DSQ-PEM), Insomnia Severity Index (ISI), Somatic Symptom Scale-8 (SSS-8), Somatic Symptom Disorder – B Criteria Scale (SSD-12), EuroQoL Five-Dimension Five-Level Questionnaire (EQ-5D-5L), Pain Disability Index (PDI), Primary Care PTSD Screen for DSM-5 (PC-PTSD-5), PROMIS v2.0 Cognitive Function Short Form 6.

**Conclusion:**

The battery offers a selection of validated, time-efficient, and mostly free assessment tools, particularly valuable in specialized care. For routine practice, targeted partial use is recommended. The structured EMOPROM LCN selection process illustrates how clinically relevant, validated and patient-centered assessment instruments can be harmonized for multicenter research and care. PROMs hold considerable potential for improving the quality of care in post-infectious syndromes.

**Supplementary Information:**

The online version contains supplementary material available at https://doi.org/10.1186/s12955-026-02631-8.

## Introduction

Post-COVID syndrome (PCS) is a complex and heterogeneous condition characterized by a wide variety of symptoms, variable trajectories, involvement of multiple organ systems, and the lack of specific biomarkers [1, 2]. The World Health Organization (WHO) defines PCS as symptoms that persist or emerge at least three months after infection with SARS-CoV-2, and last for at least two months, with no other medically explainable cause [3]. Terminology varies across institutions: the WHO uses the term “post-COVID-19 condition” and notes that it is commonly referred to as “long COVID”, whereas the NICE guideline uses “long COVID” as an umbrella term for symptoms persisting ≥ 4 weeks and distinguishes “ongoing symptomatic COVID-19” (4–12 weeks) from “post-COVID-19 syndrome” (> 12 weeks) [4]. In the present work, we use the term PCS in alignment with the WHO definition while acknowledging the conceptual overlap with related terminology. The resulting physical and psychological burdens are often considerable and affect multiple domains of life, particularly occupational functioning [5–7]. Standardized assessment of these subjective impairments is therefore essential in order to optimize care in a targeted manner and to support consistent evaluation across healthcare settings.

Patient-reported outcome measures (PROMs) provide a structured approach to assessing symptoms, functioning and quality of life from the patient perspective. Originally developed for clinical trials, PROMs are now widely used in clinical care and research to enable prospective monitoring of individual disease trajectories, identification of predictors of persistence or improvement, and evaluation of treatment effects [8, 9]. PROMs can support patient-centered care by facilitating communication between patients and clinicians, capturing individual needs, and enabling tailored treatment strategies [10, 11]. In addition, aggregated PROMs data, e.g., at a population level, can help identify patterns or inequalities in disease progression and thus contribute to improved quality of care and health policy decision-making [6, 12]. PROMs thus not only contribute to individual care, but also provide a potential instrument for systematically advancing healthcare systems. In the context of PCS, PROMs are of particular importance, as they are often one of the few structured approaches to systematically assess patients’ subjective experiences of illness over time.

Despite a growing number of studies, there is currently no internationally consented set of validated PROMs for standardized assessment in PCS. Several systematic reviews indicate that many of the instruments in use lack sufficient validation, particularly regarding content and construct validity [7, 13, 14]. Only a few scales specifically developed for PCS, such as the *Long COVID Stigma Scale* (LCSS) [15] or the *Post-COVID Functional Status Scale* (PCFS) [16], meet the criteria for unrestricted recommendation. However, none of the existing instruments alone provides a comprehensive representation and assessment of PCS. In light of these considerations, the selection of appropriate instruments should consistently be based on established measurement theory criteria, including validity, reliability, and sensitivity to change. Moreover, the European consensus framework developed by the EURONET-SOMA group provides a structured basis for the systematic classification of PROMs within biopsychosocial domains, outcomes, mechanisms, and psychosocial risk factors [17]. In parallel, international Delphi-based initiatives have proposed core outcome measurement instruments for PCS and highlight the need for harmonization while acknowledging ongoing challenges in identifying universally accepted instruments [18].

While existing reviews provide valuable overviews of available PROMs in PCS, they primarily synthesize evidence across international contexts and focus on measurement properties. However, translating these findings into routine care and multicenter research requires additional implementation-oriented decisions that are highly context-dependent. In Germany, large-scale deployment across outpatient, inpatient, and rehabilitation settings is constrained by the availability of validated German-language versions, licensing conditions, respondent burden and feasibility within clinical workflows. Therefore, beyond evidence synthesis, an explicit harmonization process is needed to agree on a feasible and standardized battery that can be implemented consistently across centers. The present work addresses this implementation gap by reporting the structured, implementation-oriented consensus process used to translate existing measurement evidence and center-specific clinical experience into a harmonized modular PROMs battery for multicenter research and clinical care of patients with PCS in a German healthcare context. The battery is composed of established instruments with validated German-language versions and was selected to ensure feasibility for large-scale deployment across outpatient, inpatient and rehabilitation settings. Importantly, this work does not aim to provide a comprehensive inventory of all available PROMs for PCS, but rather documents a pragmatic harmonization process for multicenter implementation. The applied selection process may serve as a methodological model for integrating and harmonizing PROMs in other complex or syndrome-based clinical conditions.

## Methods

**Fig. 1:**
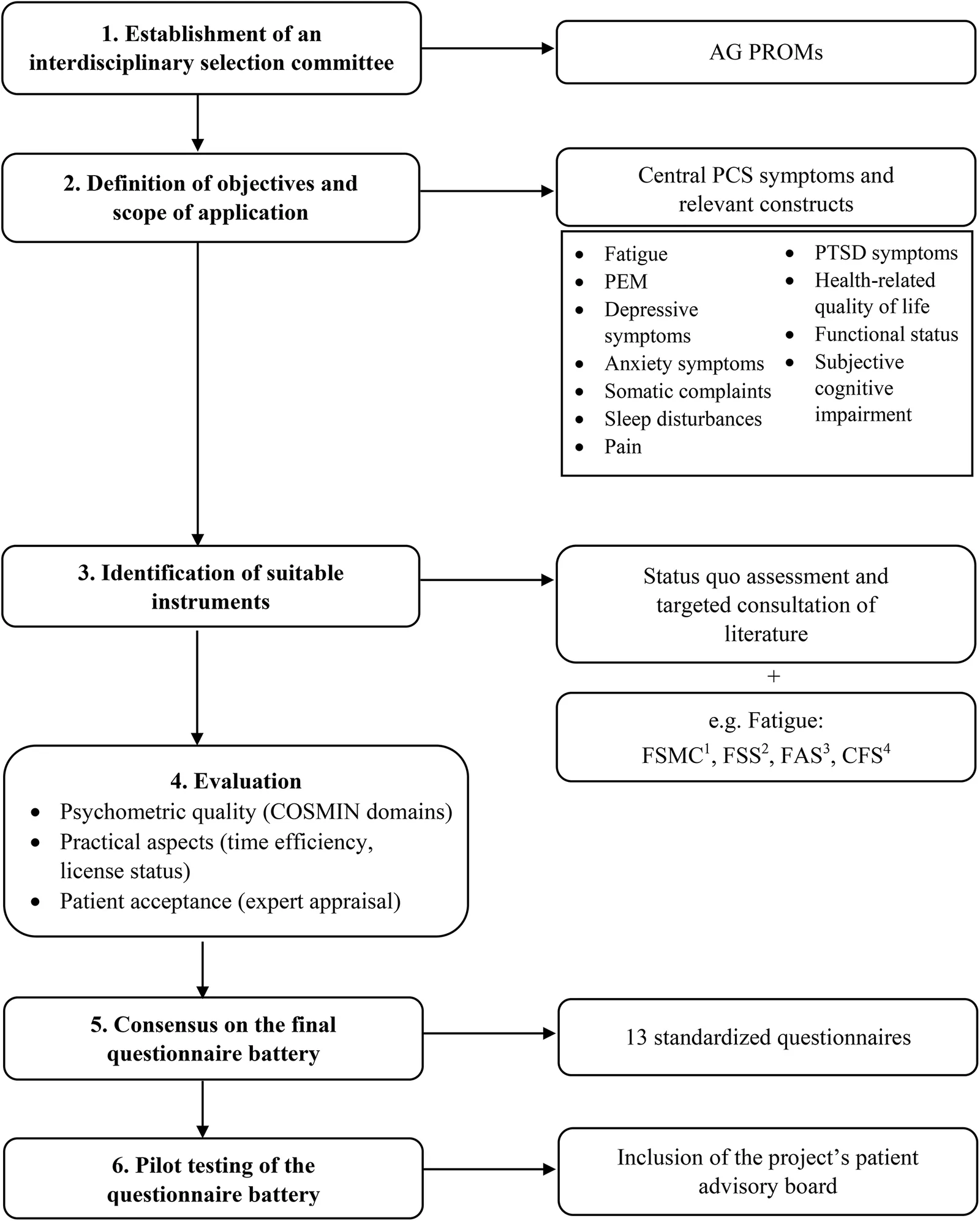
Flowchart of the consensus process in the EMOPROM LCN project. Note: ^1^FSMC = Fatigue Scale for Motor and Cognitive Functions; FSS^2^ = Fatigue Severity Scale; FAS^3^ = Fatigue Assessment Scale; CFS^4^ = Chalder Fatigue Scale. AG PROMs = working group PROMs. Pilot testing is ongoing and not reported in this manuscript

The selection of appropriate PROMs followed a multistep procedure adapted from the framework proposed by Al Sayah et al. [19] for the systematic implementation of PROMs in healthcare (see Fig. 1). The objective was to develop a modular, scientifically based, and practicable questionnaire battery for standardized assessment of relevant symptoms/syndromes and functional domains in patients with PCS, suitable for a large-scale multicenter implementation in the German healthcare context.

The EMOPROM LCN project (“Erlangen Multidisciplinary Online PROMs in the Long-COVID Network of Outpatient, Inpatient, and Rehabilitation Sectors in Collaboration with Patients”) is funded by the Federal Ministry of Health (BMG) and involves university medical centers in Erlangen, Hamburg, Ulm, and Berlin, each with long-standing, proven expertise in psychosomatics, health services research, PROMs development, as well as diagnosis and treatment of post-infectious fatigue syndromes. Alongside psychosomatic medicine, departments such as virology, neurology, general medicine, ophthalmology, immunology, and medical informatics are involved. The overarching aim of this multicenter study is to support the interdisciplinary care for patients with PCS through standardized online diagnostics based on PROMs and longitudinal monitoring.

In the first step, an internal project working group (“AG PROMs”) was established, consisting of 13 core members with minor rotation across meetings, drawn from the four participating study centers with substantial experience in PCS care, health services research, and test construction and validation. The members of the working group represented the disciplines of psychosomatic medicine, neurology, neuropsychology, rehabilitation medicine, and medical informatics. In addition to their clinical and research expertise with PROMs, they contributed practical experience from outpatient and inpatient care.

Next, the interdisciplinary working group identified the central symptom and functional domains to be covered by the PROMs battery. This step was based on current evidence on PCS symptomatology, the recommendations of the EURONET-SOMA group [17], and the participating professionals’ long-standing clinical experience in PCS care. The constructs identified as central included fatigue, post-exertional malaise (PEM), depressive symptoms, anxiety symptoms, somatic complaints, sleep disturbances, pain symptoms, health-related quality of life, functional health status, symptoms associated with post-traumatic stress disorder (PTSD), and subjectively perceived cognitive impairment.

The selection process prioritized harmonizing a core battery to be administered consistently across all participating centers. After agreeing on the key constructs, the working group compiled a cross-site overview of the instruments that had been used at the participating centers to assess these constructs. This inventory was pragmatically complemented by considering additional suitable instruments based on targeted consultation of existing reviews and key references [7, 13, 14], without conducting a formal systematic literature search. All identified candidate instruments were compiled in a shared working synopsis. Working group members from each participating center indicated which instruments were currently used at their center or preferred for implementation in the project and provided written comments on perceived advantages, limitations, and practical considerations. The completed working synopsis served as a structured basis for the subsequent consensus discussions. As a minimal methodological requirement, candidate instruments had to be supported by previously published evidence addressing content validity for the intended construct and internal consistency. Prior use within the participating centers was considered separately as an implementation-related criterion. Instruments were further evaluated using implementation-relevant considerations, including availability of validated German-language versions and cultural/linguistic appropriateness, licensing conditions, respondent burden/time efficiency, feasibility within clinical workflows, prior use in PCS contexts, and the availability of normative reference values where applicable. The appraisal of anticipated respondent burden, acceptability, and feasibility was additionally supported by several years of clinical and research experience with the selected instruments and closely comparable questionnaire batteries at the participating centers. In particular, the coordinating center in Erlangen had repeatedly administered similar batteries in a long-running study and an ongoing study. Additional published psychometric information was considered where available (e.g., reliability, construct validity, responsiveness). When several instruments were suitable for a given construct, these considerations were weighed pragmatically during the consensus discussions. Five structured consensus meetings were conducted, and meeting protocols were documented for each meeting. During these meetings, candidate instruments were discussed construct by construct using the completed working synopsis and the methodological and implementation-related criteria described above. Differing views were addressed through structured discussion of the respective advantages and limitations until a jointly acceptable selection was reached. Published psychometric evidence was weighed alongside pragmatic requirements for multicenter implementation. Decisions were reached through discussion-based expert consensus rather than through formal voting or predefined consensus thresholds. No formal Delphi procedure or nominal group technique was applied, as the process was designed as an implementation-oriented harmonization process across the participating centers. Final instrument choices for the main module were subsequently confirmed in consultation with the principal investigators of the participating centers.

The selection process was informed by established measurement theory principles, including the COSMIN taxonomy and definitions of measurement properties (e.g., reliability, validity, responsiveness) [20]. However, the procedure did not constitute a formal COSMIN-compliant systematic review of measurement properties (i.e., no COSMIN Risk of Bias checklist application and no formal evidence grading) [21].

No formal systematic literature search with predefined inclusion or exclusion criteria was conducted. Instead, the process represents a structured, expert-based consensus approach aimed at harmonizing PROMs implementation across participating centers while prioritizing validated German-language instruments suitable for planned large-scale data collection (approximately 1,000 patients).

The selected PROMs were subsequently organized thematically and transferred into a modular test battery. The main module contains instruments assessing the consented key constructs, in addition to the collection of sociodemographic and clinical data. Location-specific supplementary modules allow further research interests to be addressed, such as personality or self-efficacy. Within the EMOPROM LCN project, the web-based implementation of the battery was configured to allow participants to pause questionnaire completion at any time and resume it later, thereby limiting respondent burden. To finalize the evaluation of the questionnaire battery, the project’s patient advisory board is involved in a planned pilot phase. Patients were not members of the AG PROMs during the instrument selection phase, as the working group’s task focused on harmonizing instruments for multicenter implementation under practical constraints (e.g., validated German versions, licensing, and feasibility within clinical workflows). As part of the ongoing project activities following the initial submission, the finalized questionnaire battery was presented to the project’s patient advisory board, whose members all have lived experience of PCS. The advisory board raised no objections and did not suggest any additional content domains; aspects raised during the discussion were already covered by the proposed battery. This consultation provided a patient perspective on the proposed battery. A pilot evaluation is ongoing within the broader project and is not reported in the present manuscript.

The instrument inventory (synopsis) compiled during the consensus process is provided as Supplementary Material S1.

**Fig. 2:**
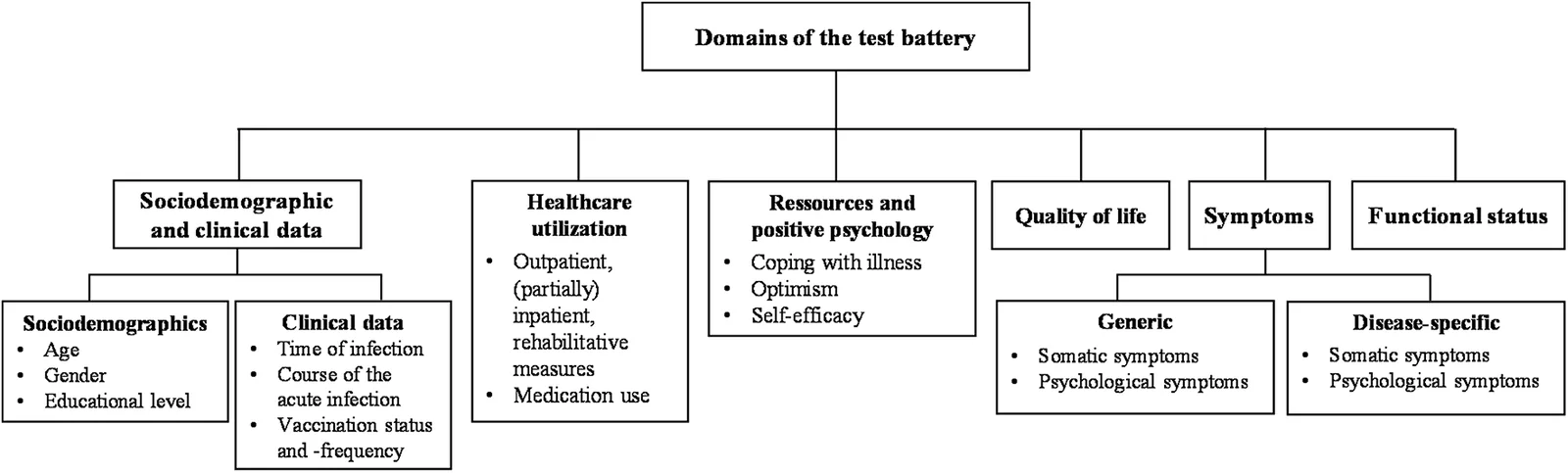
Domain structure of the EMOPROM LCN test battery

The PROMs in the final test battery were structured into domains, offering a clear thematic organization of the assessed areas. This classification reflects a comprehensive understanding of PCS and facilitates both longitudinal observation and subsequent analysis. An overview of the domain structure and the corresponding constructs is presented in Fig. 2. In addition, standardized neuropsychological test procedures and objective measures (1-minute sit-to-stand test and handgrip strength measured with a dynamometer) were included to complement the assessment of cognitive and physical functioning.

## Results

The final test battery of the main module comprises a total of 13 standardized and validated PROMs. The selection was based on the previously described evaluation of psychometric properties, practical applicability, and clinical relevance. Table 1 provides an overview of the selected PROMs, their assignment to constructs, and the key implementation-relevant considerations and selected psychometric information discussed during the expert consensus process. It does not present COSMIN ratings or a COSMIN-based evidence synthesis. The criteria shown in Table 1 were prioritized to support feasible multicenter implementation in the German healthcare context (e.g. availability of validated German-language versions, respondent burden/time efficiency, licensing, feasibility in clinical workflows), while selected psychometric information is reported where available. The reported psychometric evidence was derived from the cited validation studies and does not necessarily refer to PCS populations. “Comprehensibility for patients” reflects expert appraisal based on long-term clinical experience with these instruments in specialized outpatient and rehabilitation contexts; formal patient testing is planned within the pilot phase.

Most of the selected instruments, including the PHQ-9, GAD-7, SSS-8, SSD-12, PDI, EQ-5D-5L, and DSQ-PEM, are also included in the clinical core recommendations of the EURONET-SOMA consensus process. During the consensus process, complex considerations between scientific evidence, clinical relevance, and practical feasibility arose at several points. For the assessment of fatigue, the FSMC was selected because, unlike unidimensional scales (e.g., FSS, FAS), it captures both physical and cognitive aspects of fatigue. The PHQ-9 was included in the battery despite symptomatic overlap with post-infectious fatigue, as it provides valid assessment of depressive symptoms without making causal attributions. Furthermore, by analyzing depressive core symptoms of the PHQ-9 separately, it is possible to differentiate between PCS and indications of a depressive syndrome. A similar discussion arose regarding the assessment of somatic symptoms. Despite concerns about potential overlap with typical PCS complaints, the SSS-8 and SSD-12 were included to assess general somatic burden. The SSS-8 measures overall somatic symptom load independently of etiological concepts, while the SSD-12 additionally reflects cognitive and emotional responses to bodily symptoms. This allows for a differentiated assessment of both the extent of burden due to physical symptoms and their psychological processing.

The scope of instruments and the associated time and cognitive burden for patients were also critically evaluated in terms of feasibility. For the assessment of sleep disturbances, the ISI was chosen over the Pittsburgh Sleep Quality Index (PSQI), as it is associated with less effort to administer while offering comparable psychometric quality. Constructs involving potentially sensitive content, such as childhood trauma or PTSD symptoms, were discussed with regard to possible distress or acceptance issues. Despite individual reservations, the working group agreed to include the corresponding instruments in the supplementary module, as their scientific relevance is supported by previous findings on the significance of these factors in the chronification of somatic conditions.

Structural criteria were also taken into account. The EQ-5D-5L was included, among other reasons, due to its license-free status and international comparability. Functional health status is assessed with the PCFS, which was preferred over more general instruments such as the WHODAS due to its specific relevance and ease of use. 

**Table 1:** Overview of questionnaires included in the modular EMOPROM LCN test battery (main module)

Construct	Questionnaire	Selection criteria								
		Psychometric properties (validity, reliability) from selected evidence		Cut-off values (clinical relevance)	Validation for PCS	Time efficiency: Number of items	Sensitivity to change	License-free	Prior use in PCS	Comprehensibility for patients (expert appraisal)
Post-COVID symptoms	PCS-Score^1^ [22]	Good internal consistency (German)[22]		> 0 - ≤10.75: mild, > 10.75 - ≤26.25: moderate, > 26.25: severe[22]	✓	12	Not reported	✓	✓	✓
Functional status	PCFS^2^ [16]	Adequate content validity[13], acceptable construct validity [23]		-	✓	2 to 8	Not reported	✓	✓	✓
Fatigue	FSMC^3^ [24]	High internal consistency (α > 0.91), test-retest reliability: *r*>.80 (German)[24]		≥ 11: mild, ≥ 53: moderate, ≥ 63: severe[24]	x	20	✓	✓	✓	✓
Depressive symptoms	PHQ-9^4^ [25]	High criterion validity[26], high internal consistency (α = 0.88), very good test-retest reliability (German)[27]		≥ 10[25]	x	9	✓	✓	✓	✓
Anxiety symptoms	GAD-7^5^ [28]	Very high internal consistency (α = 0.89), high test–retest reliability (ICC=0.83) (German)[29]		≥ 10[29]	x	7	✓	✓	✓	✓
Post-exertional malaise	DSQ-PEM^6^ [30]	Good test-retest reliability, construct validity (German), good internal consistency[31]		Cut-off not validated[31, 32]	✓	10	Not reported	✓	✓	✓
Sleep disturbances	ISI^7^ [33]	Good internal consistency (α = 0.83), satisfactory test-retest reliability (*r*=.78), acceptable construct validity (German)[33]		> 10[33]	x	7	✓	✓	✓	✓
Somatic symptom burden	SSS-8^8^ [34]	High content validity, high construct validity, good reliability (α = 0.81) (German)[34]		≥ 12[34]	x	8	✓	✓	✓	✓ [34]
	SSD-12^9^ [35]	High internal consistency (α = 0.92), high construct validity (German)[35]		≥ 12[35]	x	12	✓	✓	✓	✓
Health-related quality of life	EQ-5D-5L^10^ [36]	Good interrater reliability, good test-retest reliability, good content and construct validity[14]		5 levels (1 = no problems to 5 = extreme problems)[36]	x	5	✓	✓	✓	✓
Pain	PDI^11^ [37]	Good criterion validity, high internal consistency (α = 0.86), acceptable test-retest reliability[38]		-	x	7	✓	✓	✓	✓
Post-traumatic stress disorder	PC-PTSD-5^12^ [39]	High internal consistency (α = 0.95), high test-retest reliability, high construct validity (German)[40]		> 3[39]	x	5	✓	✓	✓	✓
Subjective cognitive impairment	PROMIS v2.0 Cognitive Function^13^ [41]	High internal consistency (α = 0.95) (English)[41]		< 16th percentile[41]	x	6	Not reported	x	✓	✓

Notes. α = Cronbach’s α. 1 = Post-COVID Syndrome Score, 2 = Post-COVID Functional Status Scale, 3 = Fatigue Scale for Motor and Cognitive Functions, 4 = Patient Health Questionnaire-9, 5 = Generalized Anxiety Disorder Scale 7, 6 = DePaul Symptom Questionnaire, 7 = Insomnia Severity Index, 8 = Somatic Symptom Scale-8, 9 = Somatic Symptom Disorder – B Criteria Scale, 10 = EuroQoL Five-Dimension Five-Level Questionnaire, 11 = Pain Disability Index, 12 = Primary Care PTSD Screen for DSM-5, 13 = PROMIS v2.0 Cognitive Function Short Form 6. The reported psychometric properties were derived from the cited validation studies and were not necessarily evaluated in PCS populations. PCS-specific validation is indicated separately in the column “Validation for PCS”

## Discussion

The structured, expert-based selection and consensus process of suitable PROMs within the EMOPROM LCN project represents both a practice-oriented and methodologically founded approach to patient-centered assessment of symptoms in the context of PCS. The aim was to develop a modular PROMs battery that meets scientific requirements for measurement properties while remaining feasible under the practical conditions of clinical care. By combining EURONET-SOMA recommendations with established measurement theory principles (including COSMIN domains) and extensive clinical expertise, a harmonized set of instruments suitable for multicenter implementation was developed that enables standardized assessment of relevant health domains.

A key strength of the developed PROMs battery lies in its modular structure, which allows flexible adaptation to diverse research and clinical care contexts. The main module covers key symptom dimensions as well as quality of life and functional status, while the supplementary module includes psychosocial factors such as childhood experiences or personality. Additional objective and neuropsychological test procedures increase diagnostic validity by integrating multiple levels of assessment, particularly important in a complex condition like PCS with high subjective symptom burden. Large international cohort studies have demonstrated the multidimensional and fluctuating nature of PCS, affecting physical, cognitive and psychosocial domains simultaneously, and thereby underlining the need for multidomain core outcome assessment strategies [42]. In addition, international Delphi-based initiatives have proposed core outcome measurement instruments for PCS and emphasize the need for harmonized measurement while acknowledging ongoing uncertainty regarding optimal instruments for several outcome domains [18].

The domains and constructs covered by the PROMs battery largely align with those defined in the EURONET-SOMA consensus, ensuring broad coverage of key domains and compatibility with European consensus processes. Another strength is the interdisciplinary composition of the PROMs working group. The participation of multiple disciplines (psychosomatics, neurology, neuropsychology, rehabilitation medicine, medical informatics) enabled a comprehensive appraisal of psychometric evidence, practical feasibility, anticipated patient acceptance, and cultural appropriateness. Through the structured consensus process, a set of PROMs was selected that permits standardized yet individualized diagnostics despite heterogeneous symptom profiles in PCS.

The PROMs battery also provides a robust foundation for longitudinal assessments and data-driven treatment decisions in clinical practice. Standardized monitoring of the subjective disease course enhances communication with patients, increases transparency regarding treatment progress, and supports needs-based care [10, 11]. Evidence from other clinical fields suggests that systematic digital assessment of patient-reported outcomes in routine care can improve outcomes, including overall survival in oncology settings, highlighting the broader potential of PROMs implementation beyond symptom documentation [43].

Given the lack of specific biomarkers for PCS, the developed PROMs set contributes substantially to objectifying subjective complaints and improving diagnostic certainty. While the full battery is primarily suited for specialized care contexts such as university outpatient clinics, inpatient facilities, or rehabilitation centers, this work also offers practicing physicians the option to employ a smaller set of validated, time-efficient, and almost entirely license-free instruments. Experiences from rehabilitation clinics further suggest that PCS patients are aware of the importance of research for their treatment and therefore reliably complete even extensive questionnaire sets [44].

A relevant consideration concerns the balance between generic and disease-specific instruments. Several instruments included in the present battery (e.g., EQ-5D-5L, PHQ-9, GAD-7) are generic and were not developed specifically for PCS populations. Their inclusion was deliberate to ensure availability of validated German versions, feasibility for multicenter implementation at scale, and comparability across studies and conditions. At the same time, emerging disease-specific instruments such as the *Symptoms Evolution of Long COVID-19* (SE-LC19) illustrate advances in PROMs development using qualitative patient input and modern psychometric methods [45]. Future updates of the battery may integrate such instruments as validated German versions become available and implementation feasibility is established.

Beyond the EMOPROM LCN project, the developed PROMs selection is being integrated into broader national structures. Within the BMG-funded LongCARE initiative, a project-wide harmonization of PROMs is being pursued, with the aim of establishing a unified PROMs set to ensure comparability of patient-reported data across different Long-COVID projects in Germany. The Erlangen working group has already contributed substantially to this process on the basis of the EMOPROM LCN consensus battery. At the same time, close collaboration with medical informatics has been established to ensure interoperability of the developed PROMs set. Through the German Medical Informatics Initiative (MII), there is potential to establish PROMs as a fixed component of core data sets, enabling cross-location reuse. This will create a consolidated, standardized data structure that can support population-based PCS registries and serve as a model for other complex conditions. In the long term, this opens the possibility of integrating PROMs into electronic health records and care pathways on a broad scale, ensuring that patient-centered outcomes are routinely available for research, quality assurance, and individualized care.

Despite the structured approach, there are limitations. The selection of instruments was based on consensus decisions within the working group and is therefore partly influenced by subjective judgments. No formal systematic literature search with predefined inclusion or exclusion criteria was conducted, and the selection is therefore not intended to represent an exhaustive inventory of all available PROMs for PCS. Although informed by COSMIN measurement domains, the procedure did not imply a COSMIN-compliant systematic review of measurement properties (e.g. no Risk of Bias checklist application and no formal evidence synthesis). According to COSMIN methodology, content validity is considered the most important measurement property and ideally requires systematic qualitative evaluation involving experts and patients [46]. Our approach did not include a formal qualitative content validity study, and future research should evaluate content validity more systematically in PCS populations. The appraisal of anticipated comprehensibility, acceptability, burden, and feasibility was informed by long-standing clinical and research use of the selected instruments and closely comparable questionnaire batteries, as well as consultations with the project’s patient advisory board. However, these aspects have not yet been formally evaluated for the harmonized battery as a whole; a pilot evaluation is ongoing.

Moreover, some of the instruments have not yet been psychometrically evaluated specifically in PCS populations. In particular, longitudinal evidence on responsiveness and sensitivity to change remains limited for some instruments. Future research should therefore evaluate key measurement properties within PCS cohorts; the planned 6-month follow-up assessments in the EMOPROM LCN project provide an opportunity to examine longitudinal performance and sensitivity to change in this population. The COSMIN guideline for systematic reviews of PROMs further illustrates how measurement property evidence can be synthesized transparently; this may be a useful methodological approach for future updates of PROMs recommendations as more PCS-specific psychometric evidence accumulates [47].

In summary, the EMOPROM LCN project provides a transferable, implementation-oriented model for the structured integration of PROMs into complex care contexts. It demonstrates how scientifically well-founded, clinically relevant, and digitally implementable PROMs can contribute to improving care. The developed PROMs battery establishes a solid foundation for research and clinical practice in PCS and is also suitable for structured assessment in other complex, multidimensional conditions characterized by high subjective burden and limited objective measurability.

## Supplementary Information

Below is the link to the electronic supplementary material.

Supplementary Material 1

## Data Availability

No datasets were generated or analysed during the current study.
