# Supplementary material for: Structured, expert-informed selection and harmonization of patient-reported outcome measures (PROMs) in a German multicenter network: the post-COVID syndrome use case
Source: Health Qual Life Outcomes. 2026 Sep 25;24:126. doi: 10.1186/s12955-026-02631-8 (PMC13615613; doi:10.1186/s12955-026-02631-8)
Supplement: Supplementary file 1 [file 12955_2026_2631_MOESM1_ESM.pdf]

I. Main module

Domain	Instrument	Number of items
**Fatigue**	Fatigue Severity Scale (FSS)	*n* = 9
	Fatigue Assessment Scale (FAS)	*n* = 10
	Fatigue Scale for Motor and Cognitive Functions (FSMC)	*n* = 20
	Carruthers (BM ME/CFS Canadian Criteria for Diagnosis of ME/CFS)	7 symptom domains
**Post-exertional malaise**	DePaul Symptom Questionnaire (DSQ-PEM)	*n* = 20
**Depressive Symptoms**	PHQ-9	*n* = 9
**Anxiety symptoms**	GAD-2	*n* = 2
	GAD-7	*n* = 7
**Sleep disturbances**	Insomnia Severity Index (ISI)	*n* = 7
	Pittsburgh Sleep Quality Index (PSQI)	*n* = 20
**Somatic symptom burden**	PHQ-15	*n* = 15
	Somatic Symptom Scale-8 (SSS-8)	*n = 8*
	Somatic Symptom Disorder – B Criteria Scale (SSD-12)	*n* = 12
**Post-traumatic stress disorder**	Primary Care PTSD Screen for DSM-5 (PC-PTSD-5)	*n = 5*
**Pain**	Pain Disability Index (PDI)	*n* = 7
**Functional status**	Post COVID-19 Functional Status Scale (PCFS)	*n* = 1-4 (/4-8)
	WHODAS 2.0	*n* = 39
**Post-COVID symptoms**	PCS-Score	*n* = 12
**Health-related quality of life**	WHO-QOL	*n* = 1
	EQ-5D-5L	*n = 5*
**Subjective cognitive impairment**	PROMIS v2.0 Cognitive Function Short Form 6	*n = 6*

Note: Rows highlighted in green indicate instruments selected for inclusion in the final main module of the harmonized PROMs battery.

II. Supplementary module

Domain	Instrument	Number of items
**Work ability**	Work ability index (WAI)	*n* = 10
**Personality**	NEO-Five-Factor Inventory 30-Item-Short-Version (NEO-FFI-30)	*n* = 30
	Big Five Short Version	*n* = 10
**Achievement motive**	Achievement Motives Scale (AMS-10)	*n* = 10
**Efford-Reward-Imbalance & Overcommitment**	Efford-Reward-Imbalance Scale + Overcommitment (ERI + OC)	*n* = 16
**Coping**	Patient Competence Questionnaire (PCQ-2)	*n* = 20
	Avoidance Endurance Questionnaire (AEQ)	*n* = 49
**Social support**	ENRICHD social support inventory (ESSI)	*n* = 5
**Sense of coherence**	Sense of Coherence Scale (SOC-3)	*n* = 3
**Optimism**	Optimism-pessimism Scale (SOP2)	*n* = 1
**Religiosity**	Duke University Religion Index (DUREL)	*n* = 5
**Gratitude**	German Version of Gratitude Questionnaire-Six Item Form (GQ-5-G)	*n* = 5
**Early childhood adversities**	Childhood Trauma Questionnaire (CTQ)	*n* = 28
	Childhood Trauma Screener (CTS)	*n = 5*
